# The association of bacterial C_9_-based TTX-like compounds with *Prorocentrum minimum* opens new uncertainties about shellfish seafood safety

**DOI:** 10.1038/srep40880

**Published:** 2017-01-20

**Authors:** Inés Rodríguez, Amparo Alfonso, Eva Alonso, Juan A. Rubiolo, María Roel, Aristidis Vlamis, Panagiota Katikou, Stephen A. Jackson, Margassery Lekha Menon, Alan Dobson, Luis M. Botana

**Affiliations:** 1 Departamento de Farmacología. Facultade de Veterinaria, Universidade de Santiago de Compostela, Lugo, Spain; 2 National Reference Laboratory on Marine Toxins, Veterinary Center of Thessaloniki, Ministry of Rural Development and Food, 3A Limnou street, GR-54627 Thessaloniki, Greece; 3 School of Microbiology, University College Cork, Cork, Ireland

## Abstract

In 2012, Tetrodotoxin (TTX) was identified in mussels and linked to the presence of *Prorocentrum minimum (P. minimum*) in Greece. The connexion between TTX and *P. minimum* was further studied in this paper. First, the presence of TTX-producer bacteria, *Vibrio* and *Pseudomonas* spp, was confirmed in Greek mussels. In addition these samples showed high activity as inhibitors of sodium currents (I_Na_). *P. minimum* was before associated with neurotoxic symptoms, however, the nature and structure of toxins produced by this dinoflagellate remains unknown. Three *P. minimum* strains, ccmp1529, ccmp2811 and ccmp2956, growing in different conditions of temperature, salinity and light were used to study the production of toxic compounds. Electrophysiological assays showed no effect of ccmp2811 strain on I_Na_, while ccmp1529 and ccmp2956 strains were able to significantly reduce I_Na_ in the same way as TTX. In these samples two new compounds, *m/z* 265 and *m/z* 308, were identified and characterized by liquid chromatography tandem high-resolution mass spectrometry. Besides, two TTX-related bacteria, *Roseobacter* and *Vibrio* sp, were observed. These results show for the first time that *P. minimum* produce TTX-like compounds with a similar ion pattern and C9-base to TTX analogues and with the same effect on I_Na_.

*Prorocentrum minimum (P. minimum*) is a widely distributed neritic bloom-forming dinoflagellate first described by Pavillard in 1916. Since then, more than ten taxonomy synonyms have been used to describe the same microorganism. This controversy and confusion with names was related to the geographic area where it was isolated, including tropical, subtropical and temperate climates^1^. Blooms can occur in a wide range of environmental conditions since *P. minimum* has been described as eurythermal and euryhaline^2^.

*P. minimum* is potentially harmful to humans through consumption of toxic seafood. Over the years several human intoxications have been associated with the presence of *P. minimum* blooms. This dinoflagellate was considered responsible for more than 200 deaths in Japan, in 1942 and 1943, with symptoms such as liver injury, haemorrhage, unconsciousness, coma, and death after 24–48 hours. The toxin described in those episodes was a hydrophilic compound named venerupin and the syndrome was called Venerupin Shellfish Poisoning (VSP)^3^,^4^,^5^. At that time, *P. minimum* was considered the source of venerupin although no definitive links between dinoflagellate and toxin were reported^1^. Later on *P. minimum* was involved in several episodes of human poisoning after shellfish consumption in Portugal (Obidos Lagoon). The symptoms were considered to be characteristic of paralytic shellfish poisoning (PSP)^6^. The definitive link between *P. minimum* and neurotoxic effects was established with the analysis of several axenic clones of *P. minimum* from French coastal samples. These dinoflagellates produced a water-soluble neurotoxic compound, different from PSP, which was able to rapidly kill mice after neurotoxic symptoms^7^,^8^. This unknown compound was able to block sodium and calcium channels and was accumulated by shellfish^7^. However, since some of the toxic episodes related to *P. minimum* were also associated with the presence of other toxic microalgae such as *Dinophysis* spp., its toxicity was discussed^1^. This coexistence does not eliminate the potential risk of *P. minimum* blooms for human health, being evident that the reported toxicity of this dinoflagellate was clone-related and also related to environmental conditions^1^. In this sense, although most *P. minimum* clones were reported as non-toxic, and rare human toxicity episodes have been attributed to this specie, it should be considered for risk assessment.

Tetrodotoxin (TTX) is one of the most important neurotoxins known to block sodium channels and thus it inhibits the propagation of action potentials in muscle and nerve cells. The minimum lethal dose for TTX is 8 μg/Kg and the median lethal dose is 10 μg/Kg^9^. Symptoms can appear between 10 and 45 minutes after exposure, although depending of the amount of the toxin ingested some manifestations could appear 6 hours later. This compound blocks site 1 of voltage dependent sodium channels^10^ and it has interesting pharmacological applications in the study of excitable membranes as well as therapeutic uses in treating migraines, addictions, or as an anaesthetic agent for pain^11^.

TTX has been described in different aquatic animals such as fish, arthropods, echinoderms, molluscs, worms, newts, frogs or toads as well as several bacteria species and dinoflagellates^11^,^12^. However, the origin of TTX is still unclear, although the presence of this toxin in such a diverse group of animals suggests that bacteria might be as the primary source^13^. In this sense, TTX can be produced without bacteria, but the presence of microorganisms could, in some way, simplify TTX biosynthesis^11^.

To date, few appearances of TTX in bivalve molluscs have been reported^14^,^15^. Recently, TTX has been detected in mussels and Pacific oyster from the English coast (2013–2014)^16^. In addition in 2012, in Greece, during routine controls of shellfish, an unexplained toxicity associated with neurological symptoms was observed in a series of mouse bioassays (MBA). This atypical toxicity coincided with the absence of known dinoflagellates and other toxins, while only *P. minimum* was present in seawater. After mass-spectrometry analysis, both TTX and TTX analogues were confirmed^17^. Therefore a potential TTX presence-dinoflagellate link was hypothesized. In this context the aim of this work was to further study the relationship between TTX and *P. minimum* blooms in order to identify and characterize the compounds produced by this dinoflagellate.

## Results

To further study the occurrence and origin of TTXs in mussels collected in the unusual toxic episode in Greece, it was evaluated the presence of bacteria as TTX producers as well as the effect on Na_v_ channels.

**Figure 1:**
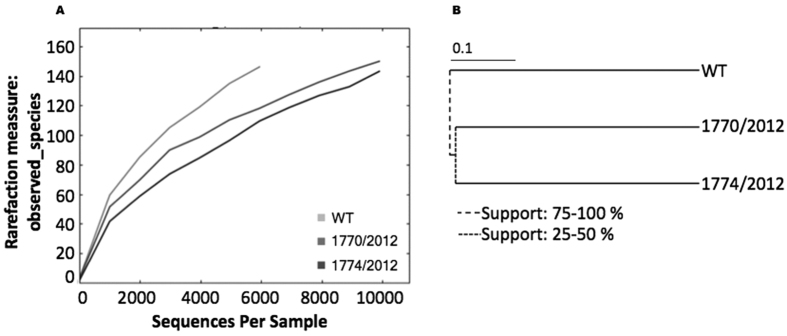
(**A**) Rarefaction curves for white tissue (WT) and TTX contaminated samples (1770/2012 and 1774/2012) samples showing the OTU based (97% 16S rRNA gene sequence similarity) number of observed species as a function of number of sequences per sample. **(B)** Bootstrapped tree for hierarchical samples clustering.

Firstly, the presence of bacteria was checked in contaminated mussel-samples, 1770/2012 and 1774/2012, from the Greek episode. Levels of 222.9 μg/Kg and 206.3 μg/Kg of TTXs respectively were reported when these samples were previously analysed by mass spectrometry^17^. Sequence reads were clustered at 97% identity resulting in 297 unique Operational Taxonomic Units (OTUs). A rarefaction plot of observed bacterial species in control (white non-contaminated tissue) and TTX-mussel samples showed higher species diversity in the uncontaminated material (Fig. 1A). Although high species diversity was achieved, uncontaminated sample was under represented when compared to TTX-mussel samples. Jackknifed hierarchical clustering by UPGMA (unweighted pair group method with arithmetic mean) showed a high support for the depurated samples clustering separate from the non-depurated one, while a lower support was observed for contaminated samples (Fig. 1B). In this way, the identified OTUs corresponded to 9 bacterial phyla (Supplementary Table 2 and Sub-Table 2). Higher percentages of proteobacterial OTUs, the phylum containing *Pseudomonas, Pseudoalteromonas*, and *Vibrio*, two known TTX producers, were detected in TTX-contaminated mussels. However these genera were not represented in the Proteobacteria of non-contaminated sample. To better identify *Vibrio* and *Pseudomonas* species, OTUs were re-picked with 100% homology. When compared to the GreenGenes 16 S RNA database^18^, several potential TTX producers were identified in both TTX-contaminated samples (Supplementary Table 3). The distribution of these bacteria showed that in both samples *Vibrio parahaemolyticus* and *Vibrio alginolyticus* were the dominant species, while *Pseudomonas* appeared at lower levels as well as other *Vibrio* species.

**Figure 2:**
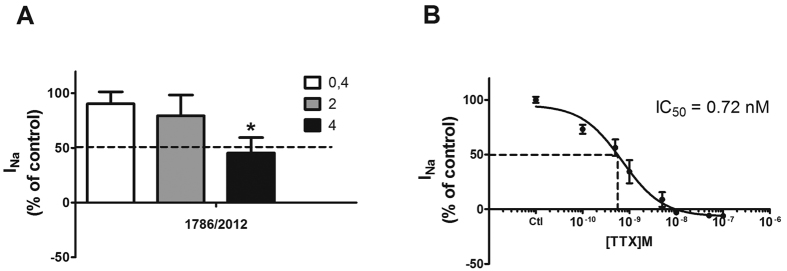
Effect of mussel sample 1786/2012 over I_Na_ current activity. Automated patch clamp experiments were carried out in hNav1.6 transfected cells. I_Na_ magnitude is expressed as percentage of basal current. **(A)** Dose-response inhibition of serial dilutions of 1786/2012 mussel sample over I_Na_. 0.4, 2 and 4 μl of extract were added to 200 μl of extracellular solution. Significant differences (*)p < 0.05. **(B)** Dose-response inhibition of TTX standard over I_Na_ with an IC_50_ of 0.43 to 1.2 nM. A discontinuous line marks the 50% of sodium current (**A** and **B**) and the corresponding TTX standard concentration (**B**).

Next, to further support the existence of TTX in mussel extracts, the effect over Na_v_ channels, the main cellular target of TTX, was checked by patch clamp measurements in stable transfected hNa_v_ 1.6 HEK cells^19^,^20^. Aliquots of 2 g of DG of sample 1786/2012 collected in Greece were extracted as previously described^17^. As Fig. 2 shows, serial dilutions of the TTX-positive mussel extract 1786/2012 were tested (0.4, 2 and 4 μl) and a dose response effect was reported. At the highest concentration tested, an inhibition of 54.65 ± 14.08% versus control current was observed (Fig. 2A). In the same cells, the control of TTX standard elicited a dose response sodium current (I_Na_) inhibition, IC_50_ 0.72 nM (95% confidence interval 0.43 to 1.2 nM) (Fig. 2B).

**Figure 3:**
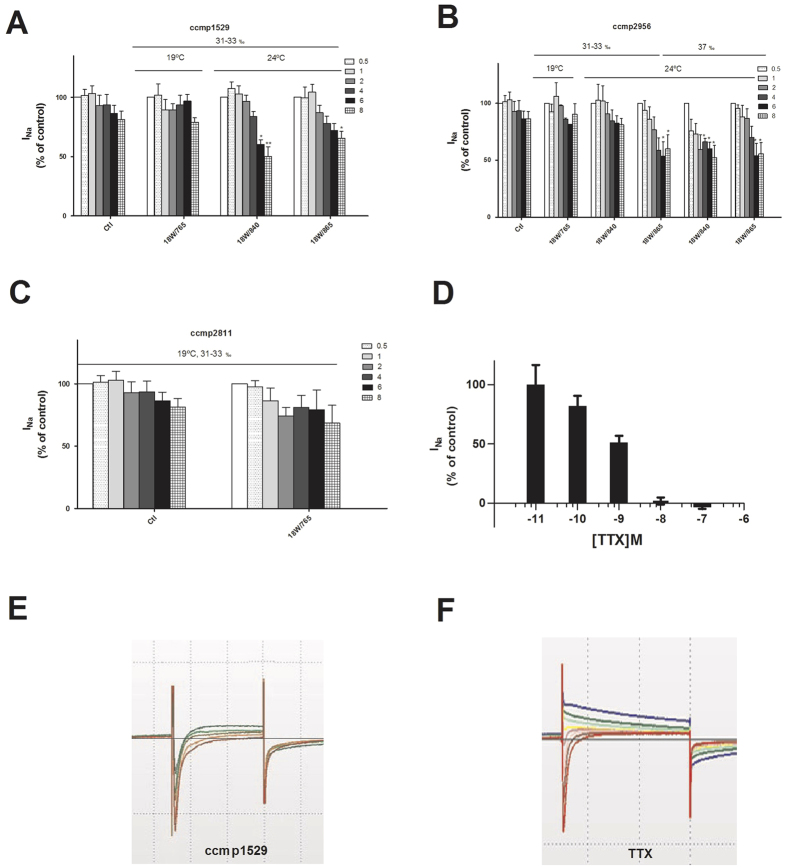
Effect of *P. minimum* extracts growing in different conditions over I_Na_ activity. Automated patch clamp experiments were carried out in hNav1.6 transfected cells. I_Na_ magnitude is expressed as percentage of basal current. **(A)** Effect of extracts from ccmp1529 strain cultured at 19 and 24 °C and different light exposures over I_Na_. **(B)** Effect of extracts from ccmp2956 strain cultured at 19 and 24 °C, two light exposures and two different salinities over I_Na_. **(C)** Effect of extracts from ccmp2811 strain cultured at 19 °C over I_Na._ (**D)** TTX dose-response inhibition of I_Na_. **(E** and **F)** Representative current traces of I_Na_ in the presence of an extract from ccmp1529 and TTX. Results are mean ± SEM of 3 experiments, each performed in duplicate. Significant differences *p < 0.05, **p < 0.01.

Although the source of TTXs in contaminated mussels seemed to be related to bacteria, the dinoflagellate *P. minimum* was present in water in high abundance when toxic mussels were harvested, therefore the presence of toxins and bacteria in this microalgae was studied. Three strains of *P. minimum* from different collection sites, ccmp1529 from Ecuador, temperature range 18 °C to 22 °C, ccmp2811 from Sarasota, Florida (USA), temperature range 18 °C to 22 °C, and ccmp2956 from Johor Strait, between Singapore and Malaysia, temperature range 22 °C to 28 °C, were cultured in L1 medium in different growth conditions, varying light intensity (785, 840 and 865), salinity (ranging from 31‰ to 37‰) and temperature (19 °C or 24 °C), with each condition being carried out in triplicate. After 15 days of growth, cells were harvested and the extracts obtained dissolved in acetic acid 0.03 M and analysed for the presence of active compounds with similar characteristics to TTXs. The same rate of growth was obtained for ccmp1529 strain either at 19 °C and 24 °C, slightly less growth was obtained when the ccmp2956 strain was grown at 19 °C compared to 24 °C and no growth was observed when the ccmp2811 strain was cultured at 24 °C. Therefore ccmp1529 and ccmp2956 strains were analysed both at 19 °C and 24 °C, while only cultures obtained at 19 °C were analysed in the case of ccmp2811. No significant effect, in terms of growth, was observed when light was modified and only ccmp2956 strain was able to grow at 37‰ of salinity. Then, the effect of dinoflagellate extracts on I_Na_ was checked. Electrophysiological recordings showed that while ccmp1529 extracts did not produce any effect on I_Na_ when dinaflagellates were grown at 19 °C, the addition of serial dilutions of 24 °C extract culture reveal a dose dependent current reduction with a maximal inhibition of 49.75 ± 8.04% *versus* control current (Fig. 3A). This effect was not dependent on light intensity. Likewise, the ccmp2956 strain cultured at 19 °C had no effect on I_Na_ but when it was grown at 24 °C and 37‰ salinity, a maximal current inhibition of 47.65 ± 10.6% *versus* control was observed (Fig. 3B). When salinity was 31–33‰, I_Na_ inhibition was dependent on light intensity, since no effect was observed with dinoflagellates cultured at 18 W/840, while 39.69 ± 12.03% inhibition was obtained at 18W/865. ccmp2811 extracts from cultures grown at 19 °C did not produce a significant I_Na_ inhibition when consecutive dilutions were added (Fig. 3C). For all these assays TTX standard was used as control of I_Na_ inhibition (Fig. 3D). Therefore, these results indicate the presence of some compound produced by *P. minimum* with a similar effect than TTX on I_Na_. The profile of compounds present in cultures grown at 19 °C or 24 °C seems to be different, at least with regard to potency over I_Na_ inhibition.

**Figure 4:**
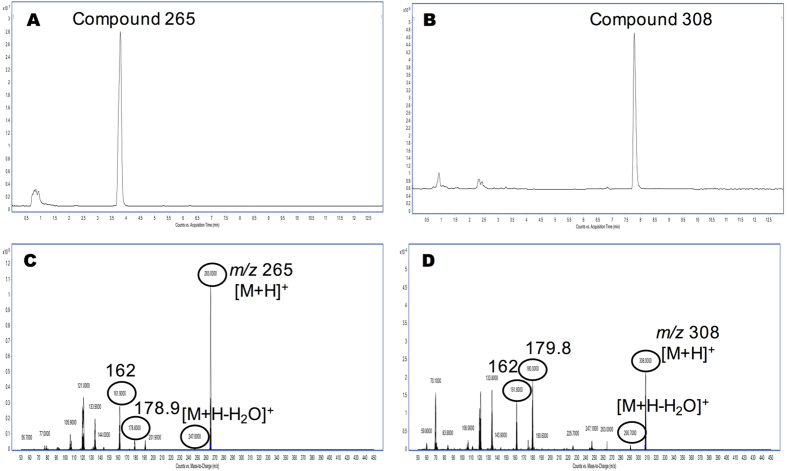
Product Ion Scan of TTX analogues *m/z* 265 and *m/z* 308 in ccmp2956 strain extract. **(A)** Product Ion Scan chromatogram obtained in positive mode of compound *m/z* 265. **(B)** Product Ion Scan chromatogram obtained in positive mode of compound *m/z* 308. **(C)** Mass spectrum obtained in Product Ion Scan of compound *m/z* 265. **(D)** Mass spectrum obtained in Product Ion Scan of compound *m/z* 308.

Next, we proceeded to the identification of compounds produced by *P. minimum* cultures using mass spectrometry technology. The first screening of culture extracts was done by Multiple Reaction Monitoring (MRM) mode, searching for the most common TTX analogues described in the literature (Supplementary Table 1). In this way, 5 compounds with same product ions, but different retention times than TTXs standards were obtained, *m/z* 320 > 302, *m/z* 304 > 162, *m/z* 302 > 162, *m/z* 290 > 272, *m/z* 272 > 254. Then, by operating in the Product Ion Scan mode, the fragmentation pathway of these five compounds was checked following the pattern fragmentation observed in TTX, 4,9-anhydroTTX and 11-deoxyTTX standards (Supplementary Figure 1B,C and D). That is, for TTX (*m/z* 320) the ions *m/z* 302 [M + H-H_2_O]^+^, *m/z* 162 and *m/z* 178 (2-aminohydroxiquinazoline and 2-aminodihydroxyquinazoline, respectively). For 4,9-anhydroTTX (*m/z* 302) the ions *m/z* 284 [M + H-H_2_O]^+^, *m/z* 162 and *m/z* 178. And in the case of 11-deoxyTTX (*m/z* 304) the ions *m/z* 286 [M + H-H_2_O]^+^, *m/z* 162 and *m/z* 176^21^. The five compounds initially observed in *P. minimum* cultures did not follow this fragmentation pathway (data not show), therefore these five compounds could not be identified as TTX analogues. However, since *P. minimum* extracts had some TTX activity, mass spectrometry operating procedure was changed from Product to Precursor Ion Scan Mode. Thus the search of compounds was addressed from the common product ion for all TTX analogues, *m/z* 162, to precursor ions. In this way, operating in the Precursor Ion Scan mode, the extract from ccmp2956 strain (24 °C and 37‰) with high inhibitory effect on I_Na_, was checked. In this sample two precursor ions, *m/z* 265 and *m/z* 308, were observed (Fig. 4). Chromatograms obtained by Ultra performance Liquid Chromatography tandem mass spectrometry (UPLC-MS/MS) show two peaks with retention times of 3.8 min and 8 min respectively (Fig. 4A and C). The Product Ion Scan of these peaks shows for the parent peak *m/z* 265 [M + H]^+^, the ions *m/z* 247 corresponding to [M + H-H_2_O]^+^, *m/z* 178.9 and *m/z* 162, Fig. 4B, and in the case of *m/z* 308 [M + H]^+^ the ions *m/z* 290 corresponding to [M + H-H_2_O]^+^, *m/z* 162 and *m/z* 179.8 (Fig. 4D).

**Figure 5:**
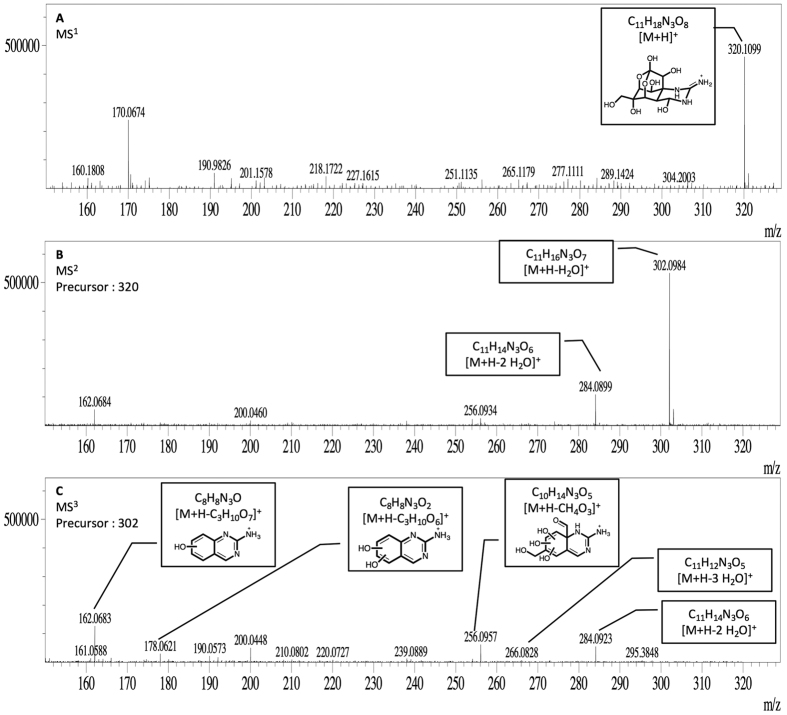
Accurate MS^1–3^ spectra of TTX standard in positive mode (*m/z* 320 and molecular formula C_11_H_17_N_3_O_8_). **(A)** MS^1^ range *m/z* 150- 50. **(B)** MS^2^ of *m/z* 320. (**C**) MS^3^ of *m/z* 302 with the predicted structures of the fragments 256, 178 and 162.

**Figure 6:**
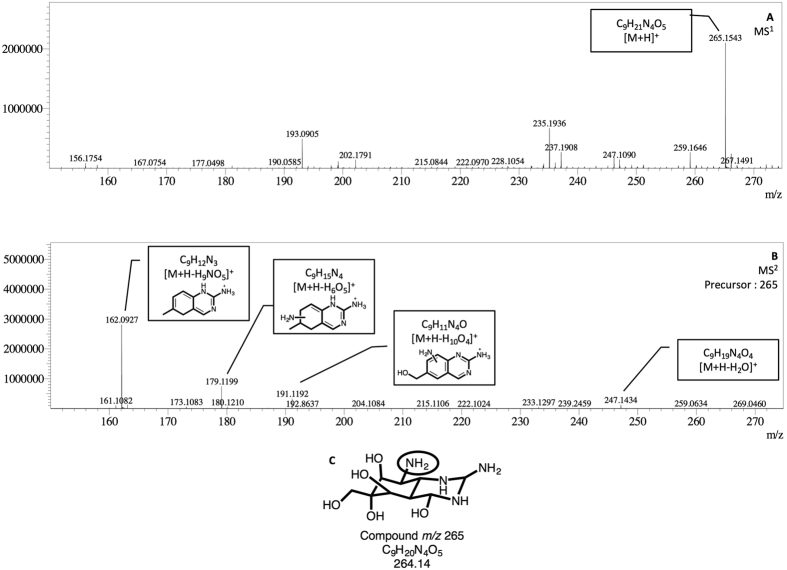
Accurate MS^1–2^ spectra of compound *m/z* 265 and molecular formula C_9_H_20_N_4_O_5_. **(A)** MS^1^ range *m/z* 150- 500. **(B)** MS^2^ of *m/z* 265 with the proposed structures of fragments 191, 179 and 162. **(C)** Proposed structure for *m/z* 265 (NH_2_ group can be in different positions).

**Figure 7:**
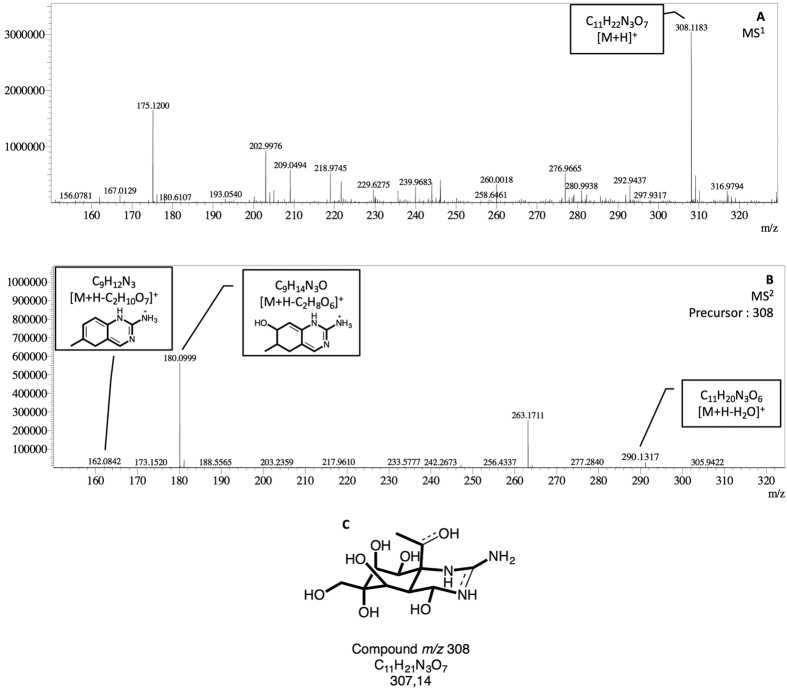
Accurate MS^1–2^ spectra of compound *m/z* 308 and molecular formula C_11_H_21_N_3_O_7_. **(A)** MS^1^ range *m/z* 150- 500. **(B)** MS^2^ of *m/z* 308 with the proposed structures of fragments 180 and 162. **(C)** Proposed structure for *m/z* 308.

To characterize and predict the molecular formula of these two new compounds, *m/z* 308 and *m/z* 265, and their product-ions, a high-resolution mass spectrometry Ion Trap-Time of Flight (IT-TOF) was used. This technology allows us to predict the formula using *ad oc* predictor software based on the accurate mass data recorded. TTX standard was employed as control to predict the molecular formula of precursor and ion products. In this way, elemental composition, accurately measured mass/charge ratio, theoretical value *m/z*, and mass errors expressed in ppm and mDa for TTX, *m/z* 308 and *m/z* 265 were obtained after MS^n^ spectra. These data are summarized in Table 1 and shown in Figs 5, 6 and 7. As Fig. 5 shows, TTX (*m/z* 320.1099, [C_11_H_17_N_3_O_8_ + H]^+^) ion was observed in MS^1^ spectrum, then in MS^2^ spectrum ions [M + H-H_2_O]^+^ at m/z 302.0984 and [M + H-2H_2_O]^+^ at m/z 284.0899 were obtained. The other characteristic ions, [M + H-C_3_H_10_O_7_]^+^ at *m/z* 162.0683 and [M + H-C_3_H_10_O_6_]^+^ at *m/z* 178.0621 and the losses 2 and 3 H_2_O molecules at *m/z* 284.0923 and *m/z* 266.0828 respectively and [M + H-CH_4_O_3_]^+^ at *m/z* 256.0957 were observed at MS^3^ spectrum. The errors between these measured ion products and the theoretical values range from 0.1–5.7 mDa, Table 1, indicating a good accuracy between the predicted molecular formula either for TTX or its ion products. Similarly, *m/z* 265 and *m/z* 308 compounds were analysed and their MS^n^ product-ions spectra are shown in Figs 6 and 7. The ion products of 265-compound (*m/z* 265.1543, [C_9_H_20_N_4_O_5_ + H]^+^) observed in MS^2^ spectrum were: [M + H-H_2_O]^+^ ion at *m/z* 247.1434, [M + H-H_10_O_4_]^+^ ion at *m/z* 191.1192, [M + H-H_6_O_5_]^+^ ion at *m/z* 179.1199, and [M + H-H_9_NO_5_]^+^ ion at *m/z* 162.0927 (Fig. 6). For the 308-compound (*m/z* 308.1183, [C_11_H_21_N_3_O_7_ + H]^+^), the ion products observed in MS^2^ spectrum were: [M + H-H_2_O]^+^ ion at *m/z* 290.1317, [M + H-C_2_H_8_O_6_]^+^ ion at *m/z* 180.0999 and [M + H-C_2_H_10_O_7_]^+^ ion at *m/z* 162.0842 (Fig. 7). Since these compounds are present in low concentration in *P. minimum* extracts only MS^2^ spectra were obtained. The errors between these measured product-ions and the theoretical values calculated are shown in Table 1. In this case the higher range observed was probably related to the low concentration. In addition the predicted structure proposed for each compound based on the high resolution masses of fragment ions are shown in Figs 5, 6 and 7.

Next, the presence of this characterized compounds, *m/z* 308 and *m/z* 265, was checked in all *P. minimum* extracts, using [M + H-H_2_O]^+^ ions for identification and *m/z* 162 ions for quantification. Since no standards of these compounds were available, results were obtained as peak area and also as ng of TTX equivalent/mL (see Supplementary Table 4). Both compounds were present in all *P. minimum* extracts. While the amount of compound 308 did not show a direct relation with the effect on I_Na_, the extracts with higher amount of compound 265 produced higher I_Na_ inhibition.

Finally, the presence of TTX-producing bacteria was checked in *P. minimum* cultures. For this experiment, ccmp1529 strain was cultured both at 19 and 24 °C, ccmp2956 strain was cultured at 19 °C, and ccmp2811 cultures were obtained at 24 °C all 31–33‰ salinity. Firstly, we checked for the presence of bacteria described in contaminated mussels, *Vibrio* and *Pseudomonas* spp. *P. minimum* cultures were analysed by PCR using specific primers for *Vibrio alginolyticus, Vibrio parahaemolyticus, Vibrio vulnificus, and Vibrio cholerae.* After PCR amplification, *V. alginolyticus* was observed in ccmp2811 and ccmp1529 cultures, while none were detected in sample ccmp2956 (Supplementary Figure 2). When the same assay was performed using specific primers for *Pseudomonas,* no positive amplification product was detected (results not shown). In addition, *P. minimum* cultures were analysed for the presence of other marine bacteria, resulting in the identification of several bacteria from *α-Proteobacterium* class, including *Roseobacter* and *Flavobacteria* (Supplementary Figure 3 and Table 5).

## Discussion

The presence of TTX in mussels is an important issue from both a toxicological and human-health risk standpoint. Although this compound is a regulated toxin in Europe and around the world, it is not included in regular monitoring programs because the consumption of species that usually accumulate the toxin is prohibited. Before 2014 only one highly toxic TTX-accumulation in scallops from Japan had been reported^14^. However, from 2014 to date three reports have identified the presence of TTX in shellfish and it was also detected in several edible gastropods^15^,^16^,^17^,^22^,^23^. Therefore, the presence of TTXs in shellfish as well as the source of these toxins should be taken into account, since TTX-contaminated samples will not be detected by official analytical methods, such as LC-MS/MS or HLPC with fluorescence detection, because TTX is not required to be included in these methods. In addition, the MBA, that could detect all toxins, is no longer used in Europe as an official assay. In this paper the presence of TTX in contaminated samples collected in Greece was confirmed through the effect on I_Na_. In addition, *V. parahaemolyticus* and *V. alginolyticus* species, two known TTX-producers, were identified in these samples^11^. Besides this, several species from *Pseudomonas* genus were also observed. This genus had previously been reported in TTX production, although the species were not specified^24^,^25^. From our data, both *Vibrio* and *Pseudomonas* sp. could be detected in TTX-contaminated mussel samples. In the case of *Pseudomonas*, several species are confirmed by 99% sequence homology and for the first time they can be clearly linked to TTX and contaminated mussels^17^.

Therefore, the connection between shellfish, bacteria and TTX in the toxic episode earlier described in Greece was clear, however, a *P. minimum* bloom was occurring at the same time and it could also be connected. Since the toxin production by this dinoflagellate has not to date been clarified, we studied its growth and toxicity in different conditions. In this way, depending on the salinity, temperature and light, *P. minimum* strains produce some compounds with an inhibitory effect on I_Na_, the same as TTX. The inhibitory effect on transfected hNa_v_ 1.6 HEK cells was higher when *P. minimum* strains from Ecuador and Malaysia were grown at 24 °C, while the Florida strain was not able to grow at this temperature. In the Malaysia strain the effect was also dependent of light and salinity conditions. These results confirm previous data where a water-soluble component produced by *P. minimum* was able to block sodium channels^7^. The variations of *P. minimum* toxicity were previously linked to the clone studied and to environmental circumstances^1^,^8^. Our results also confirm these hypotheses, since depending on the strain and on the growth conditions the effect on I_Na_ is different, as well as the toxin production.

From electrophysiological experiments with *P. minimum* extracts, the presence of some active compound produced by dinoflagellates was evident. To determine its structure, UPLC-MS/MS technology with Electrospray Ionization (ESI) source was employed to follow the fragmentation pathway of TTX and analogues earlier described^21^,^26^. In this case a column for Hydrophilic Interaction Chromatography (HILIC) with polar analytes was used and 5 compounds with apparently the same product ions but different retention times to TTX and analogues were obtained^22^,^26^,^27^. However, these compounds did not follow the characteristic fragmentation pathway of TTXs. TTX and analogues follow the same pattern, first dehydrated ions by losing a molecule of water [M + H-H_2_O], and sometimes a second one [M + H-2H_2_O], and then the *m/z* 162 and *m/z* 178 or 176 ion products. These conditions are deemed necessary to affirm that the compounds are TTX analogues^27^,^28^. Therefore, *P. minimum* extracts were checked for ion precursors of the common ion product for all TTX analogues, *m/z* 162, and two peaks *m/z* 265 and 308 were obtained. To determine its structure, high-resolution UPLC-IT-TOF technology was performed using TTX as model. Based on the predicted elemental composition and the structure of a parent compound, the structures of product ions can be obtained with a high degree of confidence^21^,^29^,^30^,^31^,^32^. As mentioned, it is well known that TTX can easily lose a molecule of water (−18 Da), yielding the abundant ion *m/z* 302. Taking into account the structure for the TTX, the loss of water can occur in different carbons but it is considered that the easiest loss is in C4^21^. This fragmentation was confirmed with a MS^3^ spectrum. In this spectrum, the most intense product ions were *m/z* 284.0877, 266.0828, 256.0957, 178.0621 and 162.0683. The product ions *m/z* 284.0877, [C_11_H_13_N_3_O_6_ + H]^+^, and 266.0828, [C_11_H_11_N_3_O_5_ + H]^+^, were formed by elimination of one (−18 Da) and two molecules of water (−36 Da), respectively. The next ion product, *m/z* 256.0957, [C_10_H_13_N_3_O_5_ + H]^+^, was formed by the elimination of CO (−28 Da) from *m/z* 284. Finally, the two last ions, *m/z* 178.0621, [C_8_H_7_N_3_O_2_ + H]^+^, and 162.0683, [C_8_H_8_N_3_O + H]^+^, were formed by the elimination of C_2_H_6_O_3_ (−78 Da) and C_2_H_6_O_4_, (−94 Da), respectively, from *m/z* 284. According to the ESI-MS^n^ results for *m/z* 265 and 308, these two compounds had similar fragmentation behaviour. Due to the small amount of compounds present in samples comparing to the TTX amount used for these experiments (2000 ng/mL), only MS^2^ could be performed and the signal intensity obtained in these spectra was low. Compound *m/z* 265, [C_9_H_20_N_4_O_5_ + H]^+^, loses a molecule of water (−18 Da) giving rise to ion *m/z* 247.1434, [C_9_H_18_N_4_O_4_ + H]^+^. The most intense product ions in MS^2^ spectra for this compound were *m/z* 191.1192, 179.1199 and 162.0927. The product ion *m/z* 191.1192, [C_9_H_10_N_4_O + H]^+^, was formed by elimination of H_10_O_4_ (−74 Da) and *m/z* 179.1199, [C_9_H_14_N_4_ + H]^+^, and *m/z* 162.0927, [C_9_H_11_N_3_ + H]^+^ by elimination of 86 and 104 Da respectively. Compound *m/z* 308 [C_11_H_21_N_3_O_7_ + H]^+^ loses a molecule of water, *m/z* 290.1317, [C_11_H_19_N_3_O_6_ + H]^+^. Besides, *m/z* 180.0999, [C_9_H_13_N_3_O + H]^+^, and *m/z* 162.0842, [C_9_H_12_N_3_ + H]^+^, are also obtained by elimination of C_2_H_8_O_6_ and of C_2_H_10_O_7_ respectively. The ion *m/z* 162.0662 identified, as product ion of TTX is different from *m/z* 162.1026 identified as product ion *m/z* 265 and *m/z* 308. In the first case it is a C8 molecule while in the second it is a C9. These structures and molecular formula were also proposed as fragment ions of 5,6,11-trideoxyTTX^21^. In addition, a similar molecule C_9_H_9_N_3_O_2_, molecular weight 191.1, is produced after strong base treatment of TTX^15^. When the errors between theoretical and experimental data are compared some differences are observed. In the case of TTX, both precursor and product ions have a narrow mass difference (0.1–5.7 mDa). In the case of compound *m/z* 265, mass differences for precursor and 3 of 4 product ions are within the range (3.7–9.9 mDa). While in the case of compound *m/z* 308, due to the low amount, the mass differences are wider (3–26.9 mDa). Therefore the increase in error should be related to the decrease in the amount of compound used. In addition the sample were not purified, and some matrix effect is also present. However, the errors observed do not invalidate these results since the fragmentation pattern of the TTX analogues observed show the loses of water (−18 Da), *m/z* 162 and *m/z* 179–180. Therefore, *m/z* 265 and 308 compounds could be precursors, maybe important molecules for later TTXs synthesis. In this regard, bacteria associated with TTX production, *Roseobacter* genus and *V. alginolyticus* specie, have also previously been identified in *P. minimum* cultures^11^. So far, the biosynthetic pathway in organisms that produce TTX is not known and some TTX analogues have been proposed as intermediates of TTX in microorganisms^21^. Therefore, compounds *m/z* 308 and *m/z* 265 identified in *P. minimum* cultures are TTX analogues produced in or by the dinoflagellate, with the same activity on I_Na_ as TTX. These molecules could be TTX precursors in mussels.

In terms of activity it seems that the effect on Na_v_ channels is directly related to *m/z* 265 presence. However, the quantification of both molecules was done with TTX standard and can be not accurate. In addition, as happens with other TTX analogues, the effect on I_Na_ can be different^22^. In this sense, it has been described that the hydroxyl group at C6 and a CH_2_OH group in the same carbon, are necessary for TTX binding to Na_v_ channels^33^. These groups are also present in compounds *m/z* 308 and *m/z* 265. It is important to emphasize that the presence and above all the position of CH_2_OH group is important for TTX-Na_v_ channels binding, and the compounds reported in this work have the same group. Thus, although when compared to TTX structure, some groups are missing, these molecules show inhibition of I_Na_ since the C9 base of TTX is maintained. This structure is common to all TTX active analogues^21^. In this sense the C9 base of TTX is often used to determine the total amount of TTX analogues in samples^15^,^16^. However, the affinity and toxicity of analogues should be different^22^,^34^.

A symbiosis between bacteria and dinoflagellates has been often proposed, however little is known about this phenomenon. Sometimes the bacteria induce death of the algae, while in other situations it provides essential vitamins for microalgae. This is the case for *Roseobacter* sp and *P minimum* cultures, which the symbiotic association increases the production of B1 and B12 vitamins essential for the dinoflagellate^35^,^36^. *Roseobacter* sp have been associated with the production of TTX in the copepod *Pseudocaligus fugu*^37^. In this case, *m/z* 320 and *m/z* 302 ions were identified as TTX and anhydroTTX in *Roseobacter* sp. cultures. In our experiments both ions (*m/z* 320 and *m/z* 302) and other fragment ions were also detected, however since they did not follow the TTX characteristic fragmentation pathway they were not identified as TTXs. As it was reported before, TTX and analogues have been detected in many different animals and environments, in addition considerable differences were found in the analogues identified in pufferfish (TTX derivatives) and amphibians (chiquiritoxin)^21^,^38^. It has been suggested that this wide range of compounds identified is probably due to the different biosynthesis or metabolism of TTX between animals^39^. Therefore, our results point to compounds 307 (C_11_H_21_N_3_O_7_) and 264 (C_9_H_20_N_4_O_5_) as other TTX analogues produced by *P. minimum* associated with bacteria (*Roseobacter* and *Vibrio* sp). This symbiotic production may explain why the strain is not always toxic. As far as we know, this is the first time that the toxic compounds produce by *P. minimum* have been identified and related to symbiotic bacteria. The structures proposed for the C9 base in these two compounds are shown in Figs 6C and 7C. In the case of *m/z* 265, the position of the NH_2_ group is not defined. For compound *m/z* 308 two structures could be proposed because the double bond can be located in several positions. To finally clarify this, the structural NMR determination and crystallographic analysis should be performed, although this would require a massive scaling of cultures to obtain enough pure compound. A consequence of TTX being produced by bacteria in algae is that this would potentially be highly influenced by environmental conditions, hence opening a justification as to why climate change provide a link to the recent increase of TTX presence in previously unreported areas.

## Methods

### Chemicals and Solutions

TTX certified standard was purchased from Laboratorios CIFGA S.A. (Lugo, Spain). 0.5 mL of solution contains 80.7 ± 6.6 μmol TTX/Kg and 9.9 ± 1.1 μmol 4,9-anhTTX/Kg.

Acetonitrile and methanol were obtained from Panreac (Barcelona, Spain). All solvents were HPLC or analytical grade and the water was obtained from a water purification system (Milli-Q, Millipore, Spain). Formic acid was purchased from Merck (Darmstadt, Germany). Ammonium formate was from Fluka (Sigma-Aldrich, Spain). Other reagents were from Sigma (Sigma-Aldrich, Spain).

Plastic tissue cultures dishes were purchased from Falcon (Madrid, Spain). Fetal calf serum, Dulbecco’s modified Eagle medium/F12 nutrient mixture (DMED/F12), Glutamax, Minimum essential medium, non essential amino acids (MEM NEAA) and G418 were purchased from Gibco (Glasgow, UK). DetachinTM was purchased from Genlantis (USA).

### Mussel samples treatment

Aliquots of 2 g of DG (digestive glands) of sample 1786/2012 collected in Greece were extracted as previously described^17^. The extract obtained was used for patch clamp recordings.

For DNA extraction DG of samples 1770/2012 and 1774/2012^17^, and one white sample (WT) (not toxic) were separated from mussels and stored at −80 °C until processed. At least 5 DG from each sample were mechanically homogenized, and DNA was extracted using the DNA extraction kit NucleoSpin Tissue DNA, RNA and protein purification kit (Macherey Nagel, forensic quality certified free of detectable DNA). The bacterial DNA extraction protocol was used, following the manufacturer instructions. After purification DNA concentration was determined with a NanoDrop (Thermo Scientific). To study bacteria presence, 16s DNA was amplified, sequenced and analysed (See Supplementary Information).

### Automated Patch clamp electrophysiological recordings

For Na current measurements HEK-293 cells stably transfected with the Na channel hNa_v_1.6 were used^20^,^40^,^41^,^42^. This cell line was kindly provided by Dr Andrew Powell (GlaxoSmithKline R&D, Stevenage, UK). Cells were cultured in DMEM/F12 medium supplemented with 10% of fetal bovine serum, Glutamax and MEM NEAA (1% w/v). G418 was freshly added at a final concentration of 0.4 mg/ml in each cell passage. Cells were incubated in a humidified 5% CO_2_/95% air atmosphere at 37 °C until 80% of confluence. Then, cells were incubated at 30 °C for 24–48 h before electrophysiological measurements. Cells were split twice per week.

All the measurements were recorded in whole-cell patch clamp configuration using an IonFlux 16 system (Fluxion, California, USA) and the corresponding Ionflux 16 software for cell capture, seal formation, whole cell obtaining, data acquisition and analysis. After incubation at 30 °C for 24–48 h, cells were washed twice with Ca^2+^ and Mg^2+^ free phosphate buffered saline (PBS) and harvested with 5 ml of Detachin^TM^ solution. Detached cells were resuspended in extracellular solution containing (mM): 2 CaCl_2_, 1 MgCl_2_, 100 Hepes, 4 KCl, 145 NaCl, 10 TEA-Cl and 10 Glucose. pH 7.4 and 320 mOsm. Electrophysiological recordings were carried out at room temperature (±22 °C) in a 96-well IonFlux microfluidic plate^43^.

I_Na_ (Na current) was evoked by depolarizing to −10 mV for 50 ms after a 100 ms step to −120 mV from −90 mV holding potential (V_h_). The intracellular solution composition for I_Na_ recordings was (in mM): 100 CsF, 45 CsCl, 10 Hepes, 5 NaCl, 5 EGTA corrected to pH 7.1 using CsOH. The contaminating effects of resistance and capacitance currents were compensated electronically by the software. Leak resistance is measured by introducing a short 20 mV pulse at the beginning of each sweep and measuring the current difference^43^. A sampling frequency of 10 kHz was used.

### *P. minimum* cultures

Three strains of *P. minimum*, from the National Center for Marine Algae and Microbiota, Bigelow (Maine, USA) ccmp1529, ccmp2811 and ccmp2956 were incubated in L1 medium with salinity of 31–33 and 37^0^/_00_. The salinity was adjusted to these proportions by the addition of freshwater removing chlorine by aeration. The three strains were incubated at 19 °C with 16:8 h light-dark photoperiod or 24 °C with 14:10 h light-dark photoperiod and different light properties (18 W/765, 18 W/840 and 18 W/865). ccmp2811 only properly grew at 19 °C. The cells were counted using an Utermöhl camera. After 15 days, the cells were harvested by filtration and extracted with methanol. The extracts were always adjusted to 7 × 10^7^ cells/mL. Methanol extracts were vacuum dried and dissolved in acetic acid 0.03 M for further electrophysiological or spectrometry purposes. For bacteria identification, *P. minimum* cultures were harvested after 15 days growth, filtered and frozen at −80 °C until use (see bellow).

L1 medium properties: To 1 L of sterilized seawater was added: 0.075 g NaNO_3_, 0.00565 g, NaH_2_PO_4_. 2H_2_O, 1.0 mL of trace elements stock solution (1) and 1.0 mL of vitamin mix stock solution (2). (1) was made by adding (per 1 L): FeCl_3_·6H_2_O (3.15 g), Na_2_EDTA·2H_2_O (4.36 g), CuSO_4_·5H_2_O (1 × 10^−8^ M), Na_2_MoO_4_·2H_2_O (9 × 10^−8^ M), ZnSO_4_·7H_2_O (8 × 10^−8^ M), CoCl_2_·6H_2_O (5 × 10^−8^ M), MnCl_2_·4H_2_O (9 × 10^−7^ M), H_2_SeO_3_ (1 × 10^−8^ M), Na_3_VO_4_ (1 × 10^−8^ M), K_2_CrO_4_ (1 × 10^−9^ M). (2) was made by adding (per 1 L): Cyanocobalamin (0.0005 g), Thiamine HCl (0.1 g), Biotin (0.0005 g).

### UPLC Conditions

Chromatographic separation was carried out using both a 1290Infinity ultra-high-performance liquid chromatography system coupled to a 6460 Triple Quadrupole mass spectrometer (Agilent Technologies, Waldbronn, Germany) and from Shimadzu (Kyoto, Japan), two pumps (LC-30AD), autoinjector (SIL-10AC) with refrigerated rack, degasser (DGU-20A), column oven (CTO-10AS) and a system controller (SCL-10AVP) coupled to IT-TOF. The toxins were separated using a column ACQUITY UPLC BEH Amide (2.1 × 100 mm, 1.7 μm, Waters) at 35 °C. Mobile phase A was 100% water with 10 mM formic acid and 10 mM ammonium formate. Mobile phase B was acetonitrile-water (95:5), containing 5 mM formic acid and 2 mM ammonium formate. The gradient program with a flow rate of 0.4 mL/min was started with 100% B and then a linear gradient to 65% B in 7 minutes. After an isocratic hold time linear of 2 minutes at 65% B and return to the starting conditions of 100% B in 0.5 minutes. Finally, 100% B was kept for 3.5 minutes before the next injection. The injection volume was 5 μL.

### MS detection

MS detection was performed using an Agilent G6460C Triple Quadrupole mass spectrometer equipped with an Agilent Jet Stream ESI source (Agilent Technologies, Waldbronn, Germany) and an IT-TOF-MS system with an electrospray ionization (ESI) interface (Shimadzu, Kyoto, Japan). The nitrogen generator is a Nitrocraft NCLC/MC from Air Liquid (Spain).

Agilent source conditions were optimized to achieve the best sensitivity for all compounds: drying gas temperature of 250 °C and flow of 5 L/min, nebulizer gas pressure of 55 psi (Nitrocraft NCLC/MS from Air Liquid), sheath gas temperature of 400 °C and flow of 12 L/min. The capillary voltage was set to 3000 V in positive mode with a nozzle voltage of 0 V. The fragmentor was 152 and the cell accelerator voltage was 2 for each toxin in this method.

IT-TOF-MS source conditions were nebulizing gas flow, 1.5 L/min, heat block temperature and CDL temperature, 200 °C and detector voltage, 1.65 kV.

### MS/MS analysis

Initial simple analysis was done in MRM mode. The mass spectrometer was operated in positive mode and the collision energy optimized using MassHunter Optimizer software (Supplementary Table 1). Two ions product were analysed per compound, one for quantification and another for confirmation.

### Mass spectrum analysis

To confirm and identify TTX analogues the fragmentation pathway of each molecule was used. The mass spectrometer was operated in the Product Ion Scan positive mode for each TTX analogue with scan range from 50 to 350 *m/z*, scan time 1120 msec and collision energy from 20 to 50 eV. Three characteristic ions were always formed [M + H-H_2_O]^+^, *m/z* 162 and *m/z* 178 (or 176) and sometimes also [M + H-2H_2_O]^+ ^21,26,27. To confirm the presence of TTX analogues, the Precursor Ion Scan of *m/z* 162 product ion was checked with scan range from 50 to 450 *m/z*, scan time 40 msec and collision energy from 20 to 50 eV.

### IT-TOF-MS analysis

The UPLC from Shimadzu (Kyoto, Japan) mentioned above was connected with an Ion Trap-Time of Flight (IT-TOF) mass spectrometer from Shimadzu. The molecules were analysed using an ion accumulation time of 10 msec and the collision energy for MS^n^ was adjusted to 50% in the analysis and the isolation width of precursor ions was 3.0Th. For full-scan MS analyses, the spectra were recorded in the range of *m/z* 150–500. Data-dependent acquisition was set such that the most abundant ions in full-scan MS would trigger tandem mass spectrometry (MS^n^, n = 2–3). Data were acquired and processed by LC/MS solution software including a formula predictor to calculate elemental compositions.

### Formula assignments

Accurate masses of fragment ions were processed using the Lab Solution software supplied with the instrument. Any mass corresponding to the formula predictor also calculated particular compositions. To assign the elemental composition of fragment ions, the error ranges were set less than 1 Da as a limit to the calculation of possible elemental compositions using the formula predictor. The other conditions for calculating elemental compositions that were taken into account were the upper limits on the number of C, H, O, N atoms, C/H ratios and the range of double-bond-equivalent (DBE).

## Additional Information

**How to cite this article**: Rodríguez, I. *et al*. The association of bacterial C_9_-based TTX-like compounds with *Prorocentrum minimum* opens new uncertainties about shellfish seafood safety. *Sci. Rep.* **7**, 40880; doi: 10.1038/srep40880 (2017).

**Publisher's note:** Springer Nature remains neutral with regard to jurisdictional claims in published maps and institutional affiliations.

## Supplementary Material

Supplementary Information

## Figures and Tables

**Table 1:** MS^n^ Data for TTX standard and compounds *m/z* 308 and *m/z* 265.

Compound	MS^n^	Elemental composition	Theoretical value (*m/z*)	[M + H]^+^ (*m/z*)	Error (mDa)	Error (ppm)
TTX	MS^1^	C_11_H_17_N_3_O_8_	320.1088	320.1099	1.1	3.44
	MS^2^	C_11_H_15_N_3_O_7_	302.0983	302.0984	0.1	0.33
	MS^3^	C_11_H_13_N_3_O_6_	284.0877	284.0923	4.6	16.19
		C_11_H_11_N_3_O_5_	266.0771	266.0828	5.7	21.42
		C_10_H_13_N_3_O_5_	256.0928	256.0957	2.9	11.32
		C_8_H_7_N_3_O_2_	178.0611	178.0621	1.0	5.62
		C_8_H_7_N_3_O	162.0662	162.0683	2.1	12.96
*m/z* 265	MS^1^	C_9_H_20_N_4_O_5_	265.1506	265.1543	3.7	13.95
	MS^2^	C_9_H_18_N_4_O_4_	247.1401	247.1434	3.3	13.35
		C_9_H_10_N_4_O	191.0927	191.1192	26.5	138.67
		C_9_H_14_N_4_	179.1291	179.1199	−9.2	−51.36
		C_9_H_11_N_3_	162.1026	162.0927	−9.9	−61.08
*m/z* 308	MS^1^	C_11_H_21_N_3_O_7_	308.1452	308.1183	−26.9	−87.30
	MS^2^	C_11_H_19_N_3_O_6_	290.1347	290.1317	−3.0	−10.34
		C_9_H_13_N_3_O	180.1131	180.0999	−13.2	−73.29
		C_9_H_11_N_3_	162.1026	162.0842	−18.4	−113.52
