# Supplementary material for: The association of bacterial C9-based TTX-like compounds with Prorocentrum minimum opens new uncertainties about shellfish seafood safety
Source: Sci Rep. 2017 Jan 20;7:40880. doi: 10.1038/srep40880 (PMC5247728; doi:10.1038/srep40880)
Supplement: Supplementary Information [file srep40880-s1.pdf]

SUPPLEMENTARY INFORMATION

The association of bacterial C_9-based TTX-like compounds with *Prorocentrum minimum* opens new uncertainties about shellfish seafood safety

Inés Rodríguez[1], Amparo Alfonso[1], Eva Alonso[1], Juan A. Rubiolo[1], María Roel[1], Aristidis Vlamis[2], Panagiota Katikou[2], Stephen A. Jackson[3], Margassery Lekha Menon[3], Alan Dobson[3], Luis M. Botana[1,*]

[1]Departamento de Farmacología. Facultade de Veterinaria. Universidade de Santiago de Compostela.
[2]National Reference Laboratory on Marine Toxins, Veterinary Center of Thessaloniki, Ministry of Productive Reconstruction Environmental and Energy, 3A Limnou street, GR54627 Thessaloniki, Greece
[3]School of Microbiology, University College Cork, Cork, Ireland
[*]Corresponding author: luis.botana@usc.es

Methods

16s DNA amplification from mussel samples, sequencing and analysis
The hypervariable regions V1, 2, and 3 of each sample's DNA were amplified by PCR using the forward primers CCATCTCATCCCTGCGTGTCTCCGACTCAGXXXXXXXXXXAGAGTTTGAT CMTGGCTCAG (X sequence represents specific barcodes for sample identification and demultiplexing after amplification, to the left adapter sequence, to the right specific sequence for 16S DNA), and the reverse primer: B530R-
CCTATCCCCTGTGTGCCTTGGCAGTCTCAGCCGCGGCKGCTGGCAC containing the sequencing adapter plus the 16s DNA specific sequence. Specific fragments were amplified using the Advantage ® HD Polymerase Mix (Clontech, CA, USA) by 25 PCR cycles with denaturing, annealing and elongation temperatures of 94, 52, and 72°C respectively. Each sample was amplified in triplicate and amplification products were analyzed in a 2 % agarose gel by electrophoresis. Bands corresponding to the amplification products were purified from the gel using the High Pure PCR Product Purification kit (ROCHE, Spain) and amplicons from the same samples were pooled together. After purification, DNA concentration was determined using the PicoGreen® dye (Thermo Scientific, Spain). Equivalent quantities of pure amplified DNA from each sample were pooled and sequenced in 1/8 of a 454 (ROCHE, Spain) plate. For sequencing, the 454 GS-FLX Titanium system (ROCHE, Spain) was used following the manufacturer instructions. After sequencing, the reads were identified and assigned to each sample using the 454-Sequencing System Software v2.5 (ROCHE, Spain). Approximately 34000 reads for the three samples included in this work were obtained, 10294 for the control sample, 9905 for sample 1770/2012, and 14293 for sample 1774/2012 which were used to determine the microbial diversity and the presence of TTX producing bacteria in each sample.
For metagenomics analysis, the QIIME (Quantitative Insights Into Microbial Environments) [1] software was employed. This software wraps other software packages, which were used during analysis: OTUs (Operational Taxonomic Units) picking was performed with UCLUST [2]. Sequence alignment was performed with PyNAST [3], and taxonomy was assigned using the Green Genes database [4] using the RDP Classifier 2.2 [5] and BLAST [6] for classification. For specific species determination, OTU picking was performed at 100 % homology and sequences corresponding to the genus *Vibrio* and *Pseudomonas* were blasted against the Green Genes 16S RNA database [4].

DNA purification from *P. minimum* cultures and PCR amplification for *Vibrio* and *Pseudomonas* detection.
P. minimum cultures were harvested after 15 days growth, filtered and frozen at -80 °C until use. Genomic DNA was isolated from these samples using NucleoSpin® Tissue Kit (Macherey-Nagel, Duren, Germany) following the manufacturer's instructions. The concentration and purity of extracted DNA samples were quantified using a NanoDrop spectrophotometer (Thermo Fisher, Spain). DNA samples were stored at –20 °C until use.

Vibrio (*V. alginolyticus, V. parahaemolyticus, V. vulnificus, and V. cholerae*) detection was performed as previously described [7]. Targeted DNA was amplified in 50 μl reaction volumes, each containing 5 μl of Kapa Taq Buffer A, 1.5 mM MgCl2 (Kappa Biosystems, Woburn, MA), 200 μM (each) dNTP (Thermo Scientific, Spain), 1 U of KAPA Taq DNA Polymerase (Kappa Biosystems, Woburn, MA), 25 ng DNA template and a concentration of each primer set adjusted to ensure proper amplification efficiency (thermal cycling conditions: 3 min at 94 °C for initial denaturation and 30 cycles, each consisting of 30 s at 94 °C, 30 s at 60 °C, and 2 min at 72 °C, followed by a final extension step of 10 min at 72 °C). Reaction volumes were finally adjusted to 50 μl by the addition of high-performance liquid chromatography-grade H_2O. PCR reactions were performed with a PTC-100^{TM} Thermal Cycler (MJ Research, Inc., Watertown, MA).
P. aeruginosa and *Pseudomonas* spp. were screened as previously described [8,9], using the same amount of template DNA as for *Vibrio* detection. The PCR reaction concentrations were the same as for *Vibrio* and the thermal cycling conditions for *P. aeruginosa* were: 3 min at 94 °C, 25 cycles consisting of 20 s at 94 °C, 20 s at 54 °C and 40 s at 72 °C followed by a last step of 1 min at 72 °C. For *Pseudomonas* spp. the thermal cycling conditions were: 3 min at 94 °C followed by 30 thermal cycles, each consisting of 1 min at 94 °C, 1 min at 55 °C and 2 min at 72 °C. A final step of 1 min at 72 °C was also included
The amplicons were visualized by electrophoresis in a 2% agarose gel with 0.05% ethidium bromide in 0.5 M TE-buffer.

DNA purification from *P. minimum* cultures and PCR amplification for *Proteobacterium and Flavobacterium* detection.

Phenotypic characterisation: *P. minimum* samples were inoculated to agar plates on two types of bacterial growth media: (i) Starch-Yeast-Peptone Seawater Agar (10 g/L soluble starch (Sigma-Aldrich), 4 g/L yeast extract (Merck), 2 g/L peptone (Sigma-Aldrich), 33.3 g/L Instant Ocean [10] (Aquarium Systems– Blacksburg, VA, USA), 15 g/L agar (Sigma-Aldrich) and (ii) LB Agar (20 g/L).

Extraction and Quantification of Total DNA: Total genomic DNA was isolated from the samples, adapted with a slight modification [11]. The sample was initially washed twice with artificial sea water (33.3 g/L Instant Ocean) and then centrifuged for 5 min at 1400Xg in an Eppendorf 5417D microfuge. The pellet was suspended in 800 µL of TENPvpp Buffer (50 mM Tris Borate (Sigma-Aldrich), 20 mM EDTA (Sigma-Aldrich), 100 mM NaCl (Sigma-Aldrich) and 0.01 g/L PVPP (Sigma-Aldrich). This is followed by the addition of 200 µL of 10% SDS (Sigma-Aldrich). The sample was incubated at 60 °C for 1 hr after which 125 µL of 5 M NaCl was added and mixed well. 0.3 mm glass beads of approximately 0.3 g were added and vortexed for 15 min. For efficient cell lysis, 125 µL of 10% CTAB (Sigma-Aldrich) was added and mixed well. The samples were incubated at 60°C for 15 min. An equal volume of Phenol: Chloroform: Isoamyl Alcohol was added and were mixed well by inversion and then centrifuged for 5 min at 1400Xg. The upper layer was decanted to fresh sterile Eppendorf tubes and 0.7 vol. of ice-cold isopropanol (Sigma-Aldrich) and 0.2 vol. of 3M sodium acetate (Sigma-Aldrich) of pH 5.2 were added and mixed

well. The DNA was pelleted by centrifugation at 18643Xg at 4 °C for 1 h in an Eppendorf 5417R. The DNA was then washed with 500 µL of 70% EtOH and centrifuged at 18643Xg at 4 °C for 15 min. The pellet was air-dried. The DNA was then resuspended in 50 µL of TE buffer (10 mM Tris-HCl, pH 7.5 and 1 mM EDTA, pH 8) at 55 °C. DNA concentration was measured using a NanoDrop Spectrophotometer (ND1000, ThermoFisher Scientific) and analyzed by electrophoresis on a 1% agarose gel.

PCR amplification of 16S rRNA gene: PCR was performed with a total volume of 30 µL containing sterile water, 1X Taq buffer, 0.2 mM dNTPs, template DNA, Taq DNA polymerase (0.75 U) (Thermo Scientific), and the universal primers (10 µM each) 27F (5′ AGAGTTTGATCCTGGCTCAG 3′) and 1492R (5' GGTTACCTTGTTACGACTT 3') [12]. The conditions were: initial denaturation (95 °C for 5 min), followed by 36 cycles of denaturation (94 °C for 30 sec), primer annealing (50 °C for 30 s) and primer extension (72 °C for 2 min), completed with a final primer extension step (72 °C for 10 min). PCR products were then visualized by electrophoresis on a 1% agarose gel.

Cloning and Sequencing of PCR amplicons: PCR products were purified using the Qiagen Kit according to the manufacturer's instructions. The products were then cloned using the Qiagen PCR Cloning Kit as provided by the manufacturer. PCR amplicons were ligated to pDRIVE vector and cloned into EZ Competent cells provided by Qiagen. The cells were plated on to LB plates containing Kanamycin (30 µg/ml), IPTG (50 µM) and X-Gal (80 µg/mL). The recombinants were screened by blue/white selection.

M13 PCR: The transformants were lysed by boiling at 98 °C for 15 min in 100 µL TE buffer. The mixture was then centrifuged at 18643Xg for 15 min at 4 °C and 3 µL of the supernatant was used as a template DNA for the PCR. PCR was performed with a total volume of 30 µL containing sterile water, 1X Taq buffer, 2 mM dNTPs, template DNA, Taq DNA polymerase (0.75 U) (Thermo Scientific), primers (10 µM each) M13F (5'-GTAAAACGACGGCCAGT-3') and M13R (5'- GTTTTCCCAGTCACGAC-3'). The conditions were as follows: initial denaturation (94 °C for 5 min), followed by 36 cycles of denaturation (94 °C for 30 s), primer annealing (50°C for 30 s) and primer extension (72 °C for 90 s), completed with a final primer extension step (72 °C for 10 min). PCR products were then analyzed by 1% agarose gel.

Sequencing and Analysis of the PCR amplicons: PCR products were purified using the Qiagen Kit according to the instructions provided by the manufacturer. The PCR products were sequenced by GATC Biotech, UK by Sanger sequencing (single extension capillary sequencing). Sequences were trimmed for quality using FinchTV (http://www.geospiza.com/ftvdlinfo.html). Sequence similarity searches were performed using the BLASTn suite program available at the NCBI (http://blast.ncbi.nlm.nih.gov/Blast.cgi).

Figures

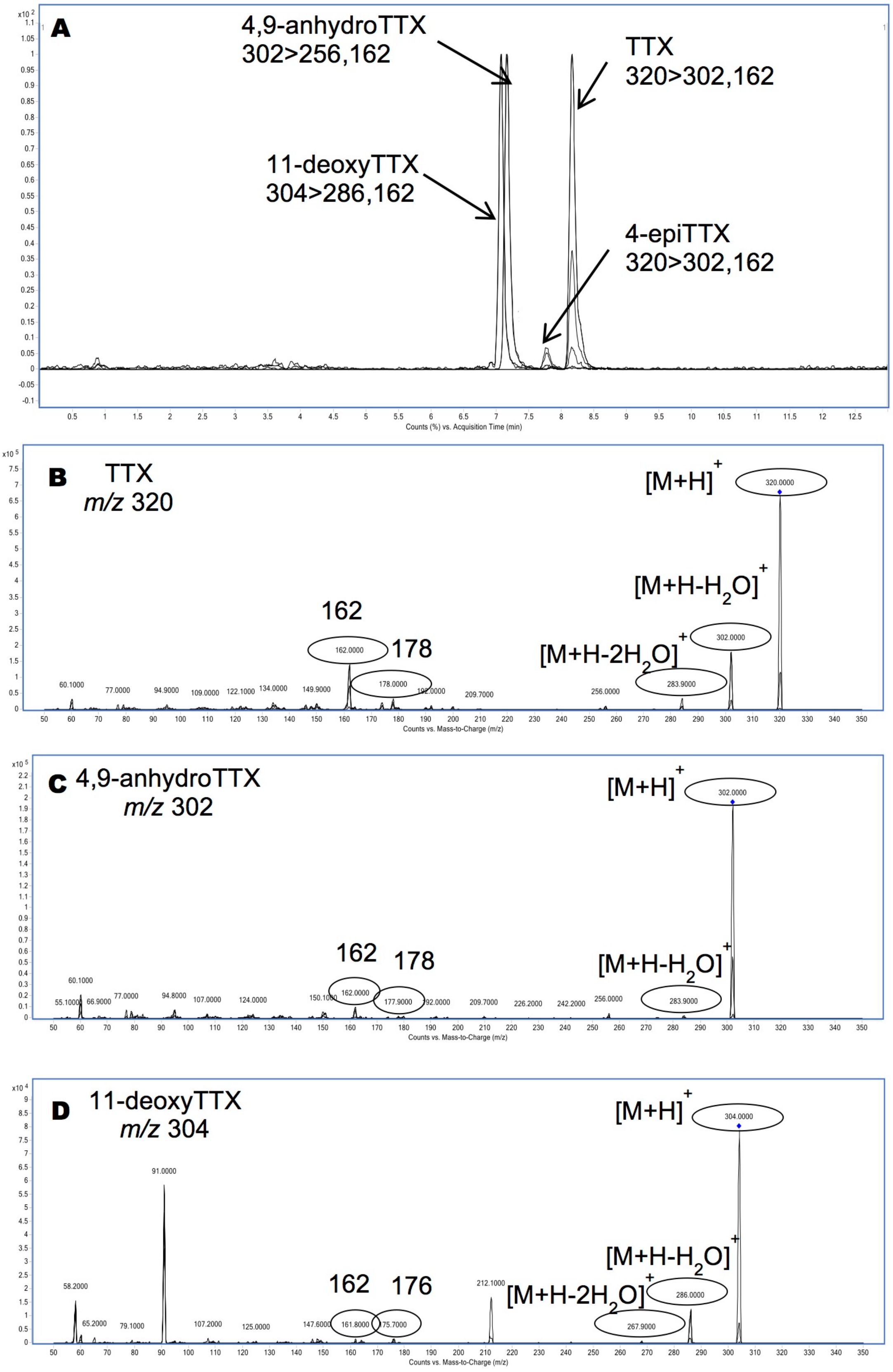

A
4,9-anhydroTTX 302>256,162
TTX 320>302,162
11-deoxyTTX 304>286,162
4-epiTTX 320>302,162
Counts (%) vs. Acquisition Time (min)
B
TTX m/z 320
[M+H]+
[M+H-H2O]+
[M+H-2H2O]+
162
178
Counts vs. Mass-to-Charge (m/z)
C
4,9-anhydroTTX m/z 302
[M+H]+
[M+H-H2O]+
162
178
Counts vs. Mass-to-Charge (m/z)
D
11-deoxyTTX m/z 304
[M+H]+
[M+H-H2O]+
[M+H-2H2O]+
162
176
Counts vs. Mass-to-Charge (m/z)

SI Figure 1. TTX standard analysis. (A) MRM chromatogram obtained in positive mode of TTX standard (1000 ng/mL), transitions of 320>302 m/z and 320>161.9 m/z (TTX), 302>256 m/z and 302>161.9 m/z (4,9-anhydroTTX), 304>286 m/z and 304>176 m/z (11-deoxyTTX). **(B)** Mass spectrums obtained in Product Ion Scan mode of TTX *m/z* 320. **(C)** Mass spectrums obtained in Product Ion Scan mode of 4,9-anhydroTTX *m/z* 302. **(D)** Mass spectrums obtained in Product Ion Scan mode of 11-deoxyTTX *m/z* 304.

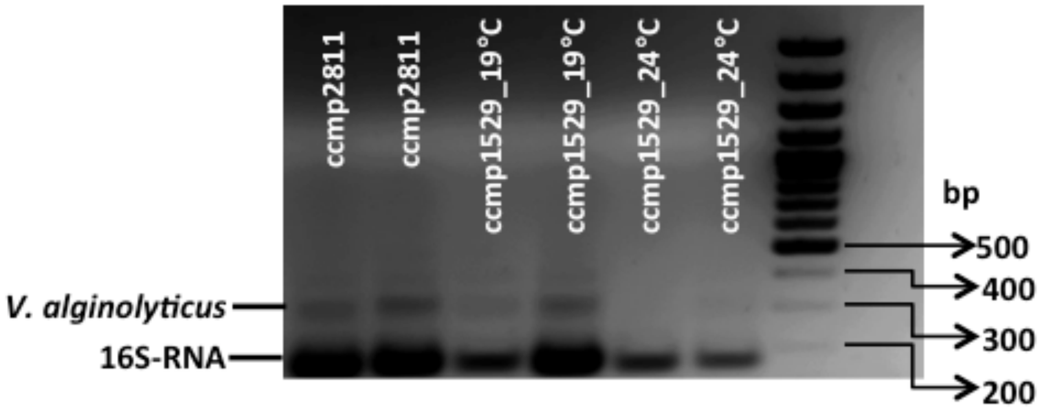

SI Figure 2: Multiplex PCR amplification of genomic DNA in ccmp2811 and ccmp1529 strains. *V. parahaemolyticus, V. alginolyticus, V. vulnificus* and *V. cholerae* bacteria analysis.

A

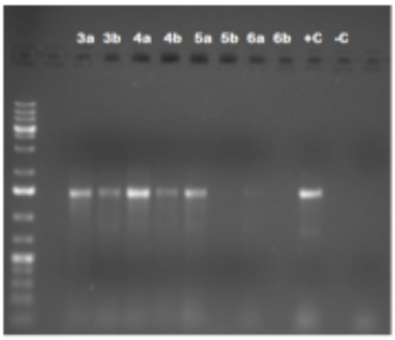

B

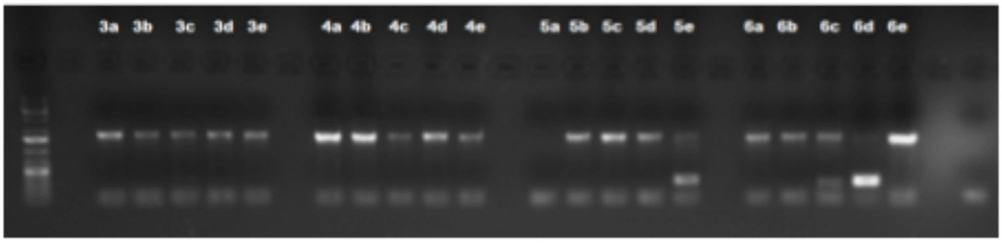

SI Figure 3. (A) 16S PCR from ccmp1529 strain DNA extracts. ccmp1529 grown at 19 °C (3a, 3b, 4a and 4b) and at 24 °C (5a, 5b, 6a and 6b). **(B)** M13 PCR of cloned 16S PCR products, ccmp1529 grown at 19 °C (3a, 3b, 3c, 3d, 3e, 4a, 4b, 4c, 4d and 4e) and at 24 °C (5a, 5b, 5c, 5d, 5e, 6a, 6b, 6c, 6d and 6e).

Tables

Compound Name	Precursor Ion	Product Ion	Collision Energy
**11-oxo-TTX**	336	318<br>300<br>282<br>178<br>162	24<br>24<br>36<br>40<br>40
**TTX**<br>**4-epiTTX**<br>**6-epiTTX**<br>**Tetrodonic acid**	320	302<br>161,9	24<br>36
**5-deoxyTTX**<br>**11-deoxyTTX**	304	286<br>176	24<br>40
**4,9-anhydro-TTX**<br>**6-epi-4,9-anhydroTTX**<br>**anhydroTTX**	302	256<br>161,9	28<br>40
**11-norTTX-6(R)-ol**<br>**11-norTTX-6(S)-ol**	290	272<br>162	24<br>40
**6,11-dideoxyTTX**	288	270<br>224	24<br>40
**1-hydroxy-8-epi-5,6,11-trideoxyTTX**	288	270<br>162	24<br>40
**5,6,11-trideoxyTTX**<br>**8-epi-5,6,11-trideoxyTTX**	272	254<br>162	24<br>40
**1-hydroxy-4,4a-anhydro-8-epi-5,6,11-trideoxyTTX**	270	252<br>162	24<br>40
**4,-anhydro-8-epi-5,6,11-trideoxyTTX**	254	236<br>162<br>81	24<br>40<br>40

Table 1. Precursor and product ions (*m/z*) of TTX and analogues and MS conditions.

Taxon[1]	WT[2]	1770/2012[2]	1774/2012[2]
Unassigned;Other	0.228	0.615	0.4557246
k__Bacteria; p__Actinobacteria	0.016	0.050	0.04907803
k__Bacteria; p__Bacteroidetes	0.000	0.000	0.02804459
k__Bacteria; p__Cyanobacteria	0.016	0.010	0.0140223
k__Bacteria; p__Firmicutes	99.560	85.916	92.5681834
k__Bacteria; p__Fusobacteria	0.000	0.000	0.11918951
k__Bacteria; p__Planctomycetes	0.032	0.050	0.02103344
k__Bacteria; p__Proteobacteria	0.146	13.356	6.723
k__Bacteria; p__Tenericutes	0.000	0.000	0.014
k__Bacteria; p__Verrucomicrobia	0.000	0.000	0.007

[1]Phylum level.
[2]Values are presented as percentages.

SI Table 2: OTUs abundance in mussel samples: control (WT) and TTX-positive 1770/2012 and 1774/2012.

Taxon[3]	WT[2]	1770/2012[2]	1774/2012[2]
k__Bacteria; p__Proteobacteria; c_Gammaproteobacteria; o_Pseudomonadales; f_Pseudomonadaseae	0.000	0.01	0.316
k__Bacteria; p__Proteobacteria; c_Gammaproteobacteria; o_Vi bionales; f_Pseudoalteromonadaceae	0.000	2.797	3.449
k__Bacteria; p__Proteobacteria; c_gammaproteobacteria; o_Vi bionales;f_Vibrionaceae	0.000	2.917	1.970

[3]Up to Family level

SI Sub-table 2: OTUs of potential TTX producers: From k__Bacteria; p__Proteobacteria

**1770/2012**			
Bacterial Genera, Species and Strain[1]	Number of Sequences[2]	Query cover (%)	Ident (%)
Vibrio parahaemolyticus[3] strain ATCC 17802<br>*Vibrio parahaemolyticus*[3] strain O3:K6	154	99-100	99
Vibrio alginolyticus[3] strain NBRC 15630<br>*Vibrio alginolyticus*[3] strain ATCC 17749	269	99-100	99
Vibrio tubiashii strain Milford 74<br>*Vibrio nereis* strain 80<br>*Vibrio hippocampi* strain BFLP-4<br>*Vibrio celticus* strain Rd 8.15<br>*Vibrio artabrorum* strain VB 11.8	8	98-100	94-99
Vibrio rumoiensis strain S-1	6	95	98
Vibrio tapetis strain CECT 4600	3	99	98
Pseudomonas[4] *extremaustralis* strain 14-3<br>*Pseudomonas*[4] *veronii* strain CIP 104663<br>*Pseudomonas*[4] *poae* RE*1-1-14 strain RE*1-1-14<br>*Pseudomonas*[4] *tolaasii* strain NCPPB 2192<br>*Pseudomonas*[4] *chlororaphis* subsp. *aureofaciens* strain DSM 6698<br>*Pseudomonas*[4] *orientalis* strain CFML 96-170<br>*Pseudomonas*[4] *fluorescens* strain CCM 2115<br>*Pseudomonas*[4] *chlororaphis* subsp. *aurantiaca* strain NCIB 10068	3	99	99
**1774/2012**			
Vibrio parahaemolyticus[3] RIMD 2210633<br>*Vibrio parahaemolyticus*[3] train ATCC 17802	395	99-100	99
Vibrio alginolyticus[3] strain ATCC 17749<br>*Vibrio alginolyticus*[3] strain NBRC 15630	368		
Vibrio fischeri ES114[3] strain ES114	20	100	99
Vibrio rumoiensis strain S-1	10	95	96-98
Vibrio aestuarianus subsp. francensis strain 02/041<br>*Vibrio aestuarianus* strain OY 0-002	9	99	94-97
Vibrio nereis strain 80<br>*Vibrio natriegens* strain ATCC 14048<br>*Vibrio pelagius* strain ATCC 25916	20	100	97-99
Pseudomonas[4] *cuatrocienegasensis* strain 1N<br>*Pseudomonas*[4] *taeanensis* strain MS-3<br>*Pseudomonas*[4] *gessardii* strain CIP 105469<br>*Pseudomonas*[4] *libanensis* strain CIP 105460<br>*Pseudomonas*[4] *poae* RE1-1-14 strain RE1-1-14<br>*Pseudomonas*[4] *extremaustralis* strain 14-3	51	99-100	98-99

Pseudomonas[4] *veronii* strain CIP 104663<br>*Pseudomonas*[4] *tolaasii* strain NCPPB 2192			

[1]Determined by BLASTn using the partial 16S rRNA sequenced. When more than one species is included in a row, means that we could not one from another by sequence homology for a particular OTU. [2]Identified after assignment of taxonomy using a 100 % similarity. [3]Bacteria which have been shown to produce TTX. [4]Genus which has been shown to produce TTX. For a comprehensive review to TTX producing bacteria see [13].

SI Table 3: *Vibrio* and *Pseudomonas* species present in TTX-contaminated mussel samples.

	***m/z* 308**		***m/z* 265**	
	**Peak area**	**ng TTX equivalent /mL**	**Peak area**	**ng TTX equivalent /mL**
**ccmp1529 18W/765 19 °C**	155519	51.62	350088	112.89
**ccmp1529 18W/840 24 °C**	78518	27.38	1769954	560.03
**ccmp1529 18W/865 24 °C**	124360	41.81	1222463	387.62
**ccmp2956 18W/765 19 °C**	251106	60.02	102231	65.05
**ccmp2956 18W/840 24 °C**	240608	78.42	122061	41.05
**ccmp2956 18W/865 24 °C**	265768	86.34	736056	234.44
**ccmp2956 18W/840 24 °C**	251604	81.88	2084631	665.98
**ccmp2956 18W/865 24 °C**	350540	70.05	563869	317.23
**ccmp2811 18W/765 19 °C**	37592	14.36	329141	106.30

SI Table 4: Quantification of compounds, *m/z* 308 and *m/z* 265, in *P. minimum* cultures.

CLONE ID	BLAST RELATIVE OF CLONED 16S rRNA GENE
3a	*Marivita litorea* strain CL-JM1 NR_044513 (99% sequence identity)
3b	*Marivita litorea* strain CL-JM1 NR_044513 (99% sequence identity)
3 c	*Marivita* sp. DG1517 KC295393 (100% sequence identity)
3 d	*Marivita litorea* strain CL-JM1 NR_044513 (99% sequence identity)
3 e	*Roseobacter* sp. SYOP1 DQ659418 (99% sequence identity)
4 a	*Roseobacter* sp. SYOP1 DQ659418 (99% sequence identity)
4 b	*Roseobacter* sp. SYOP1 DQ659418 (99% sequence identity)
4 c	*Mameliella alba* strain JLT354-W EU734592 (99% sequence identity)
4 d	*Gilvibacter sediminis* strain Mok-1-36 NR_041451 (99% sequence identity)
4 e	*Roseobacter* sp. SYOP1 DQ659418 (99% sequence identity)
5 b	*Gilvibacter* sp. MOLA 433 AM990700 (100% sequence identity)
5 c	*Gilvibacter sediminis* Mok-1-36 NR_041451 (99% sequence identity)
5 d	*Gilvibacter* sp. MOLA 433 AM990700 (100% sequence identity)
6 b	*Gilvibacter* sp. MOLA 433 AM990700 (100% sequence identity)
6 e	*Gilvibacter* sp. MOLA 433 AM990700 (100% sequence identity)

SI Table 5: BLAST analysis of cloned 16S PCR products of ccmp1529 extracts. ccmp1529 grown at 19 ºC (3a, 3b, 3c, 3d, 3e, 4a, 4b, 4c, 4d and 4e) and at 24 ºC (5b, 5c 6b, and 6e).

References

1 Caporaso, J. G. *et al.* QIIME allows analysis of high-throughput community sequencing data. *Nature methods* **7**, 335-336, doi:10.1038/nmeth.f.303 (2010).

2 Edgar, R. C. Search and clustering orders of magnitude faster than BLAST. *Bioinformatics* **26**, 2460-2461, doi:10.1093/bioinformatics/btq461 (2010).

3 Caporaso, J. G. *et al.* PyNAST: a flexible tool for aligning sequences to a template alignment. *Bioinformatics* **26**, 266-267, doi:10.1093/bioinformatics/btp636 (2010).

4 DeSantis, T. Z. *et al.* Greengenes, a Chimera-Checked 16S rRNA Gene Database and Workbench Compatible with ARB. *Appl Environ Microbiol* **72**, 5069-5072 (2006).

5 Wang, Q., Garrity, G. M., Tiedje, J. M. & Cole, J. R. Naive Bayesian classifier for rapid assignment of rRNA sequences into the new bacterial taxonomy. *Appl Environ Microbiol* **73**, 5261-5267, doi:10.1128/AEM.00062-07 (2007).

6 Altschul, S. F., Gish, W., Miller, W., Myers, E. W. & Lipman, D. J. Basic local alignment search tool. *Journal of molecular biology* **215**, 403-410, doi:10.1016/S0022-2836(05)80360-2 (1990).

7 Wei, S., Zhao, H., Xian, Y., Hussain, M. A. & Wu, X. Multiplex PCR assays for the detection of Vibrio alginolyticus, Vibrio parahaemolyticus, Vibrio vulnificus, and Vibrio cholerae with an internal amplification control. *Diagnostic microbiology and infectious disease* **79**, 115-118, doi:10.1016/j.diagmicrobio.2014.03.012 (2014).

8 Karpati, F. & Jonasson, J. Polymerase chain reaction for the detection of Pseudomonas aeruginosa, Stenotrophomonas maltophilia and Burkholderia cepacia in sputum of patients with cystic fibrosis. *Molecular and cellular probes* **10**, 397-403, doi:10.1006/mcpr.1996.0055 (1996).

9 Spilker, T., Coenye, T., Vandamme, P. & LiPuma, J. J. PCR-based assay for differentiation of Pseudomonas aeruginosa from other Pseudomonas species recovered from cystic fibrosis patients. *Journal of clinical microbiology* **42**, 2074-2079 (2004).

10 Atkinson, M. J. & Bingman, C. Elemental composition of commercial seasalts. *J. Aquaric. Aquat. Sci.* **8**, 39-43 (1998).

11 Shan, G., Jin, W., Lam, E. K. & Xing, X. Purification of total DNA extracted from activated sludge. *Journal of environmental sciences* **20**, 80-87 (2008).

12 Turner, S., Pryer, K. M., Miao, V. P. & Palmer, J. D. Investigating deep phylogenetic relationships among cyanobacteria and plastids by small subunit rRNA sequence analysis. *The Journal of eukaryotic microbiology* **46**, 327-338 (1999).

13 Jal, S. & Khora, S. S. An overview on the origin and production of tetrodotoxin, a potent neurotoxin. *Journal of applied microbiology* **119**, 907-916, doi:10.1111/jam.12896 (2015).
